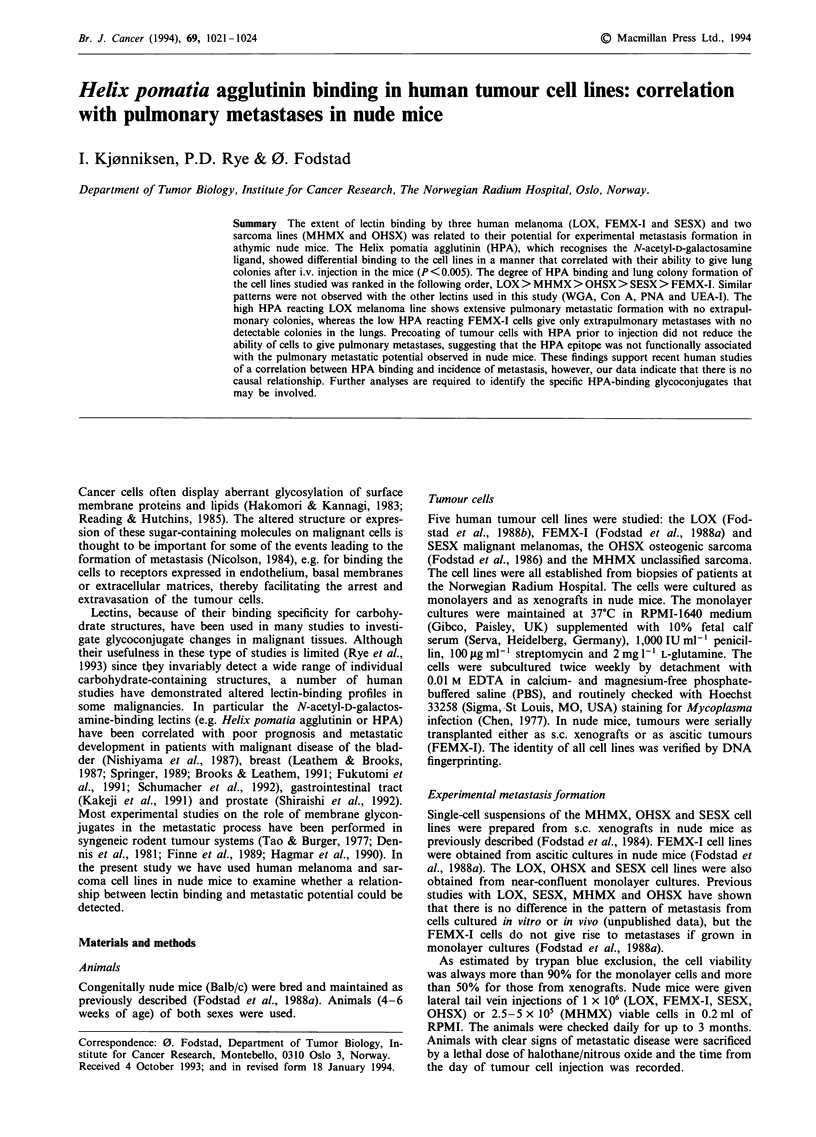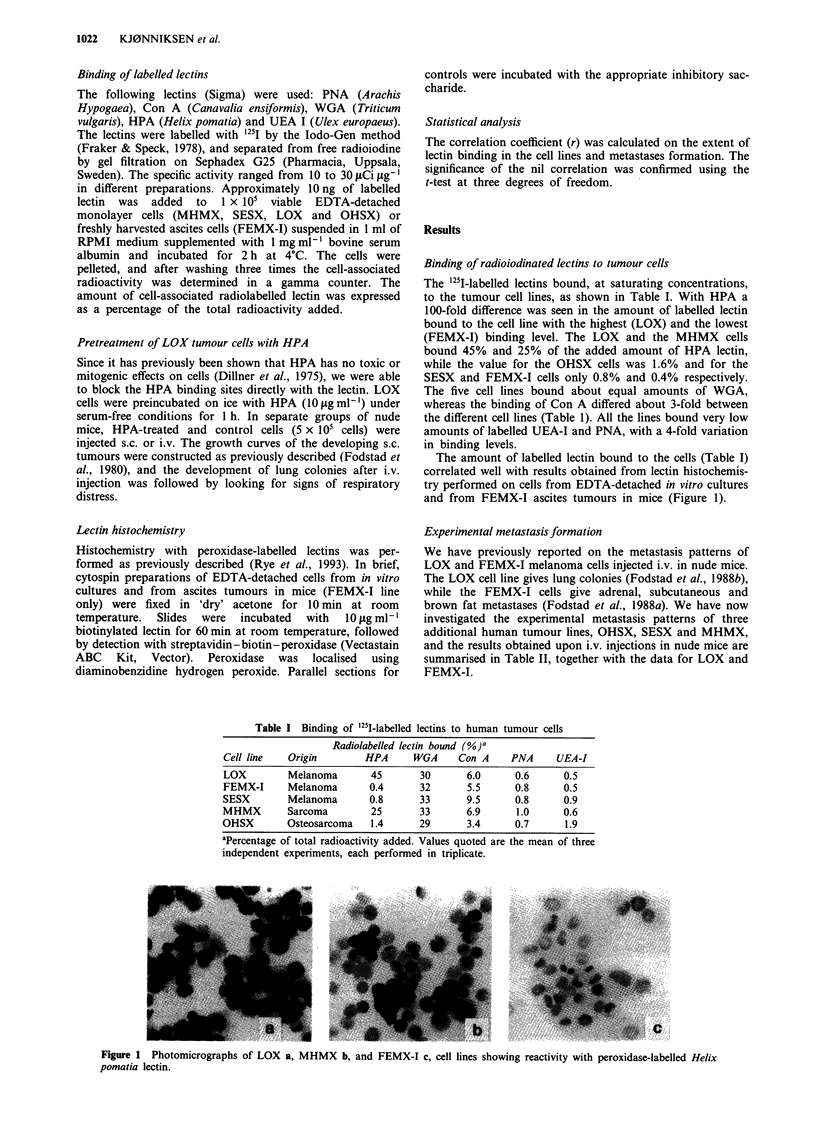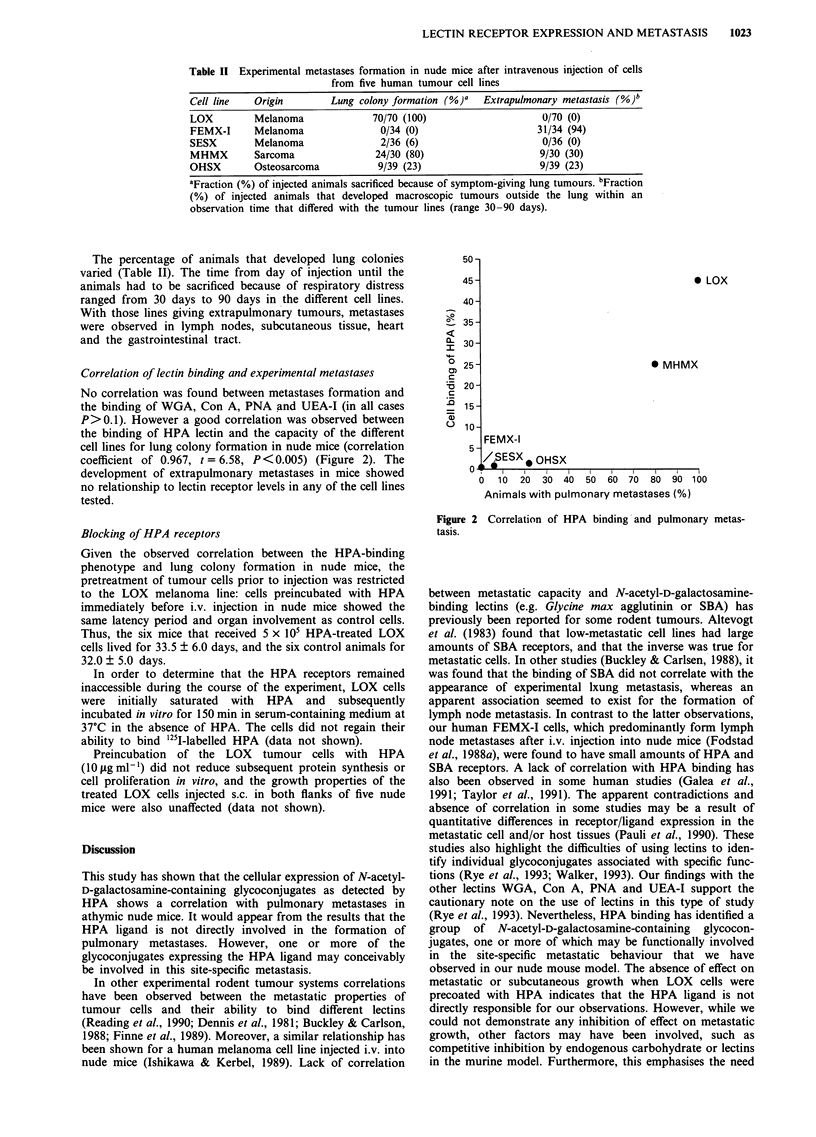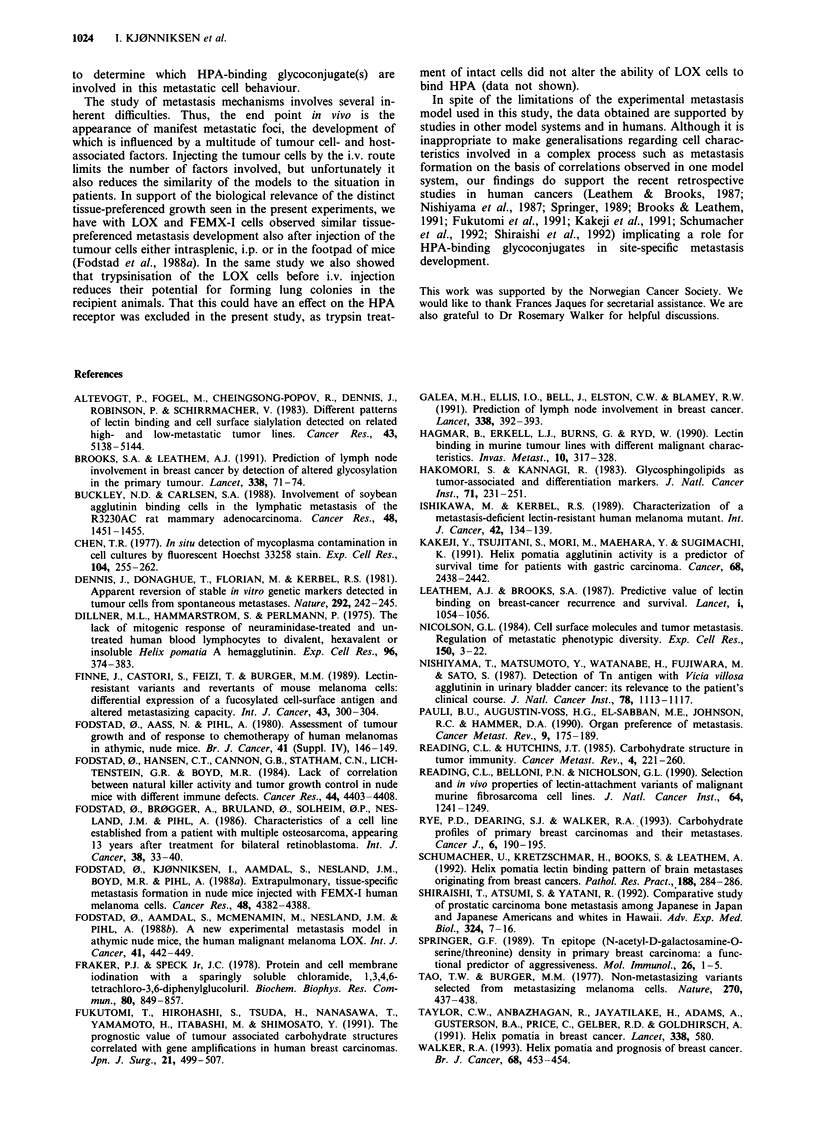# Helix pomatia agglutinin binding in human tumour cell lines: correlation with pulmonary metastases in nude mice.

**DOI:** 10.1038/bjc.1994.200

**Published:** 1994-06

**Authors:** I. Kjønniksen, P. D. Rye, O. Fodstad

**Affiliations:** Department of Tumor Biology, Norwegian Radium Hospital, Oslo.

## Abstract

**Images:**


Br. J. Cancer (1994), 69, 1021-1024 © Macmillan Press Ltd., 1994

### *Helix pomatia* agglutinin binding in human tumour cell lines: correlation with pulmonary metastases in nude mice

I. Kjønniksen, P.D. Rye & Ø. Fodstad

*Department of Tumor Biology, Institute for Cancer Research, The Norwegian Radium Hospital, Oslo, Norway.*

**Summary** The extent of lectin binding by three human melanoma (LOX, FEMX-I and SESX) and two sarcoma lines (MHMX and OHSX) was related to their potential for experimental metastasis formation in athymic nude mice. The Helix pomatia agglutinin (HPA), which recognises the *N*-acetyl-D-galactosamine ligand, showed differential binding to the cell lines in a manner that correlated with their ability to give lung colonies after i.v. injection in the mice ($P < 0.005$). The degree of HPA binding and lung colony formation of the cell lines studied was ranked in the following order, LOX > MHMX > OHSX > SESX > FEMX-I. Similar patterns were not observed with the other lectins used in this study (WGA, Con A, PNA and UEA-I). The high HPA reacting LOX melanoma line shows extensive pulmonary metastatic formation with no extrapulmonary colonies, whereas the low HPA reacting FEMX-I cells give only extrapulmonary metastases with no detectable colonies in the lungs. Precoating of tumour cells with HPA prior to injection did not reduce the ability of cells to give pulmonary metastases, suggesting that the HPA epitope was not functionally associated with the pulmonary metastatic potential observed in nude mice. These findings support recent human studies of a correlation between HPA binding and incidence of metastasis, however, our data indicate that there is no causal relationship. Further analyses are required to identify the specific HPA-binding glycoconjugates that may be involved.

Cancer cells often display aberrant glycosylation of surface membrane proteins and lipids (Hakomori & Kannagi, 1983; Reading & Hutchins, 1985). The altered structure or expression of these sugar-containing molecules on malignant cells is thought to be important for some of the events leading to the formation of metastasis (Nicolson, 1984), e.g. for binding the cells to receptors expressed in endothelium, basal membranes or extracellular matrices, thereby facilitating the arrest and extravasation of the tumour cells.

Lectins, because of their binding specificity for carbohydrate structures, have been used in many studies to investigate glycoconjugate changes in malignant tissues. Although their usefulness in these type of studies is limited (Rye *et al.*, 1993) since they invariably detect a wide range of individual carbohydrate-containing structures, a number of human studies have demonstrated altered lectin-binding profiles in some malignancies. In particular the *N*-acetyl-D-galactosamine-binding lectins (e.g. *Helix pomatia* agglutinin or HPA) have been correlated with poor prognosis and metastatic development in patients with malignant disease of the bladder (Nishiyama *et al.*, 1987), breast (Leathem & Brooks, 1987; Springer, 1989; Brooks & Leathem, 1991; Fukutomi *et al.*, 1991; Schumacher *et al.*, 1992), gastrointestinal tract (Kakeji *et al.*, 1991) and prostate (Shiraishi *et al.*, 1992). Most experimental studies on the role of membrane glycoconjugates in the metastatic process have been performed in syngeneic rodent tumour systems (Tao & Burger, 1977; Dennis *et al.*, 1981; Finne *et al.*, 1989; Hagmar *et al.*, 1990). In the present study we have used human melanoma and sarcoma cell lines in nude mice to examine whether a relationship between lectin binding and metastatic potential could be detected.

### Materials and methods

### *Animals*

Congenitally nude mice (Balb/c) were bred and maintained as previously described (Fodstad *et al.*, 1988*a*). Animals (4-6 weeks of age) of both sexes were used.

Correspondence: Ø. Fodstad, Department of Tumor Biology, Institute for Cancer Research, Montebello, 0310 Oslo 3, Norway.
Received 4 October 1993; and in revised form 18 January 1994.

### *Tumour cells*

Five human tumour cell lines were studied: the LOX (Fodstad *et al.*, 1988*b*), FEMX-I (Fodstad *et al.*, 1988*a*) and SESX malignant melanomas, the OHSX osteogenic sarcoma (Fodstad *et al.*, 1986) and the MHMX unclassified sarcoma. The cell lines were all established from biopsies of patients at the Norwegian Radium Hospital. The cells were cultured as monolayers and as xenografts in nude mice. The monolayer cultures were maintained at 37°C in RPMI-1640 medium (Gibco, Paisley, UK) supplemented with 10% fetal calf serum (Serva, Heidelberg, Germany), 1,000 IU $ml^{-1}$ penicillin, 100 $\mu g\ ml^{-1}$ streptomycin and 2 $mg\ l^{-1}$ L-glutamine. The cells were subcultured twice weekly by detachment with 0.01 M EDTA in calcium- and magnesium-free phosphate-buffered saline (PBS), and routinely checked with Hoechst 33258 (Sigma, St Louis, MO, USA) staining for *Mycoplasma* infection (Chen, 1977). In nude mice, tumours were serially transplanted either as s.c. xenografts or as ascitic tumours (FEMX-I). The identity of all cell lines was verified by DNA fingerprinting.

### *Experimental metastasis formation*

Single-cell suspensions of the MHMX, OHSX and SESX cell lines were prepared from s.c. xenografts in nude mice as previously described (Fodstad *et al.*, 1984). FEMX-I cell lines were obtained from ascitic cultures in nude mice (Fodstad *et al.*, 1988*a*). The LOX, OHSX and SESX cell lines were also obtained from near-confluent monolayer cultures. Previous studies with LOX, SESX, MHMX and OHSX have shown that there is no difference in the pattern of metastasis from cells cultured *in vitro* or *in vivo* (unpublished data), but the FEMX-I cells do not give rise to metastases if grown in monolayer cultures (Fodstad *et al.*, 1988*a*).

As estimated by trypan blue exclusion, the cell viability was always more than 90% for the monolayer cells and more than 50% for those from xenografts. Nude mice were given lateral tail vein injections of $1 \times 10^6$ (LOX, FEMX-I, SESX, OHSX) or $2.5-5 \times 10^5$ (MHMX) viable cells in 0.2 ml of RPMI. The animals were checked daily for up to 3 months. Animals with clear signs of metastatic disease were sacrificed by a lethal dose of halothane/nitrous oxide and the time from the day of tumour cell injection was recorded.

*Binding of labelled lectins*

The following lectins (Sigma) were used: PNA (*Arachis Hypogaea*), Con A (*Canavalia ensiformis*), WGA (*Triticum vulgaris*), HPA (*Helix pomatia*) and UEA I (*Ulex europaeus*). The lectins were labelled with $^{125}I$ by the Iodo-Gen method (Fraker & Speck, 1978), and separated from free radioiodine by gel filtration on Sephadex G25 (Pharmacia, Uppsala, Sweden). The specific activity ranged from 10 to 30 $\mu Ci\ \mu g^{-1}$ in different preparations. Approximately 10 ng of labelled lectin was added to $1 \times 10^5$ viable EDTA-detached monolayer cells (MHMX, SESX, LOX and OHSX) or freshly harvested ascites cells (FEMX-I) suspended in 1 ml of RPMI medium supplemented with 1 mg $ml^{-1}$ bovine serum albumin and incubated for 2 h at 4°C. The cells were pelleted, and after washing three times the cell-associated radioactivity was determined in a gamma counter. The amount of cell-associated radiolabelled lectin was expressed as a percentage of the total radioactivity added.

*Pretreatment of LOX tumour cells with HPA*

Since it has previously been shown that HPA has no toxic or mitogenic effects on cells (Dillner *et al.*, 1975), we were able to block the HPA binding sites directly with the lectin. LOX cells were preincubated on ice with HPA (10 $\mu g\ ml^{-1}$) under serum-free conditions for 1 h. In separate groups of nude mice, HPA-treated and control cells ($5 \times 10^5$ cells) were injected s.c. or i.v. The growth curves of the developing s.c. tumours were constructed as previously described (Fodstad *et al.*, 1980), and the development of lung colonies after i.v. injection was followed by looking for signs of respiratory distress.

*Lectin histochemistry*

Histochemistry with peroxidase-labelled lectins was performed as previously described (Rye *et al.*, 1993). In brief, cytospin preparations of EDTA-detached cells from *in vitro* cultures and from ascites tumours in mice (FEMX-I line only) were fixed in 'dry' acetone for 10 min at room temperature. Slides were incubated with 10 $\mu g\ ml^{-1}$ biotinylated lectin for 60 min at room temperature, followed by detection with streptavidin-biotin-peroxidase (Vectastain ABC Kit, Vector). Peroxidase was localised using diaminobenzidine hydrogen peroxide. Parallel sections for controls were incubated with the appropriate inhibitory saccharide.

*Statistical analysis*

The correlation coefficient ($r$) was calculated on the extent of lectin binding in the cell lines and metastases formation. The significance of the nil correlation was confirmed using the $t$-test at three degrees of freedom.

### Results

*Binding of radioiodinated lectins to tumour cells*

The $^{125}I$-labelled lectins bound, at saturating concentrations, to the tumour cell lines, as shown in Table I. With HPA a 100-fold difference was seen in the amount of labelled lectin bound to the cell line with the highest (LOX) and the lowest (FEMX-I) binding level. The LOX and the MHMX cells bound 45% and 25% of the added amount of HPA lectin, while the value for the OHSX cells was 1.6% and for the SESX and FEMX-I cells only 0.8% and 0.4% respectively. The five cell lines bound about equal amounts of WGA, whereas the binding of Con A differed about 3-fold between the different cell lines (Table 1). All the lines bound very low amounts of labelled UEA-I and PNA, with a 4-fold variation in binding levels.

The amount of labelled lectin bound to the cells (Table I) correlated well with results obtained from lectin histochemistry performed on cells from EDTA-detached *in vitro* cultures and from FEMX-I ascites tumours in mice (Figure 1).

*Experimental metastasis formation*

We have previously reported on the metastasis patterns of LOX and FEMX-I melanoma cells injected i.v. in nude mice. The LOX cell line gives lung colonies (Fodstad *et al.*, 1988*b*), while the FEMX-I cells give adrenal, subcutaneous and brown fat metastases (Fodstad *et al.*, 1988*a*). We have now investigated the experimental metastasis patterns of three additional human tumour lines, OHSX, SESX and MHMX, and the results obtained upon i.v. injections in nude mice are summarised in Table II, together with the data for LOX and FEMX-I.

**Table I** Binding of $^{125}I$-labelled lectins to human tumour cells

| | | Radiolabelled lectin bound (%)[a] | | | | |
|---|---|---|---|---|---|---|
| *Cell line* | *Origin* | *HPA* | *WGA* | *Con A* | *PNA* | *UEA-I* |
| LOX | Melanoma | 45 | 30 | 6.0 | 0.6 | 0.5 |
| FEMX-I | Melanoma | 0.4 | 32 | 5.5 | 0.8 | 0.5 |
| SESX | Melanoma | 0.8 | 33 | 9.5 | 0.8 | 0.9 |
| MHMX | Sarcoma | 25 | 33 | 6.9 | 1.0 | 0.6 |
| OHSX | Osteosarcoma | 1.4 | 29 | 3.4 | 0.7 | 1.9 |

[a]Percentage of total radioactivity added. Values quoted are the mean of three independent experiments, each performed in triplicate.

**Figure 1** Photomicrographs of LOX **a**, MHMX **b**, and FEMX-I **c**, cell lines showing reactivity with peroxidase-labelled *Helix pomatia* lectin.

**Table II** Experimental metastases formation in nude mice after intravenous injection of cells from five human tumour cell lines

| *Cell line* | *Origin* | *Lung colony formation (%)*[a] | *Extrapulmonary metastasis (%)*[b] |
|---|---|---|---|
| LOX | Melanoma | 70/70 (100) | 0/70 (0) |
| FEMX-I | Melanoma | 0/34 (0) | 31/34 (94) |
| SESX | Melanoma | 2/36 (6) | 0/36 (0) |
| MHMX | Sarcoma | 24/30 (80) | 9/30 (30) |
| OHSX | Osteosarcoma | 9/39 (23) | 9/39 (23) |

[a]Fraction (%) of injected animals sacrificed because of symptom-giving lung tumours. [b]Fraction (%) of injected animals that developed macroscopic tumours outside the lung within an observation time that differed with the tumour lines (range 30–90 days).

The percentage of animals that developed lung colonies varied (Table II). The time from day of injection until the animals had to be sacrificed because of respiratory distress ranged from 30 days to 90 days in the different cell lines. With those lines giving extrapulmonary tumours, metastases were observed in lymph nodes, subcutaneous tissue, heart and the gastrointestinal tract.

*Correlation of lectin binding and experimental metastases*

No correlation was found between metastases formation and the binding of WGA, Con A, PNA and UEA-I (in all cases $P > 0.1$). However a good correlation was observed between the binding of HPA lectin and the capacity of the different cell lines for lung colony formation in nude mice (correlation coefficient of 0.967, $t = 6.58$, $P < 0.005$) (Figure 2). The development of extrapulmonary metastases in mice showed no relationship to lectin receptor levels in any of the cell lines tested.

**Figure 2** Correlation of HPA binding and pulmonary metastasis.

*Blocking of HPA receptors*

Given the observed correlation between the HPA-binding phenotype and lung colony formation in nude mice, the pretreatment of tumour cells prior to injection was restricted to the LOX melanoma line: cells preincubated with HPA immediately before i.v. injection in nude mice showed the same latency period and organ involvement as control cells. Thus, the six mice that received $5 \times 10^5$ HPA-treated LOX cells lived for $33.5 \pm 6.0$ days, and the six control animals for $32.0 \pm 5.0$ days.

In order to determine that the HPA receptors remained inaccessible during the course of the experiment, LOX cells were initially saturated with HPA and subsequently incubated *in vitro* for 150 min in serum-containing medium at 37°C in the absence of HPA. The cells did not regain their ability to bind $^{125}$I-labelled HPA (data not shown).

Preincubation of the LOX tumour cells with HPA ($10\ \mu g\ ml^{-1}$) did not reduce subsequent protein synthesis or cell proliferation *in vitro*, and the growth properties of the treated LOX cells injected s.c. in both flanks of five nude mice were also unaffected (data not shown).

### Discussion

This study has shown that the cellular expression of *N*-acetyl-D-galactosamine-containing glycoconjugates as detected by HPA shows a correlation with pulmonary metastases in athymic nude mice. It would appear from the results that the HPA ligand is not directly involved in the formation of pulmonary metastases. However, one or more of the glycoconjugates expressing the HPA ligand may conceivably be involved in this site-specific metastasis.

In other experimental rodent tumour systems correlations have been observed between the metastatic properties of tumour cells and their ability to bind different lectins (Reading *et al.*, 1990; Dennis *et al.*, 1981; Buckley & Carlson, 1988; Finne *et al.*, 1989). Moreover, a similar relationship has been shown for a human melanoma cell line injected i.v. into nude mice (Ishikawa & Kerbel, 1989). Lack of correlation between metastatic capacity and *N*-acetyl-D-galactosamine-binding lectins (e.g. *Glycine max* agglutinin or SBA) has previously been reported for some rodent tumours. Altevogt *et al.* (1983) found that low-metastatic cell lines had large amounts of SBA receptors, and that the inverse was true for metastatic cells. In other studies (Buckley & Carlsen, 1988), it was found that the binding of SBA did not correlate with the appearance of experimental lxung metastasis, whereas an apparent association seemed to exist for the formation of lymph node metastasis. In contrast to the latter observations, our human FEMX-I cells, which predominantly form lymph node metastases after i.v. injection into nude mice (Fodstad *et al.*, 1988*a*), were found to have small amounts of HPA and SBA receptors. A lack of correlation with HPA binding has also been observed in some human studies (Galea *et al.*, 1991; Taylor *et al.*, 1991). The apparent contradictions and absence of correlation in some studies may be a result of quantitative differences in receptor/ligand expression in the metastatic cell and/or host tissues (Pauli *et al.*, 1990). These studies also highlight the difficulties of using lectins to identify individual glycoconjugates associated with specific functions (Rye *et al.*, 1993; Walker, 1993). Our findings with the other lectins WGA, Con A, PNA and UEA-I support the cautionary note on the use of lectins in this type of study (Rye *et al.*, 1993). Nevertheless, HPA binding has identified a group of *N*-acetyl-D-galactosamine-containing glycoconjugates, one or more of which may be functionally involved in the site-specific metastatic behaviour that we have observed in our nude mouse model. The absence of effect on metastatic or subcutaneous growth when LOX cells were precoated with HPA indicates that the HPA ligand is not directly responsible for our observations. However, while we could not demonstrate any inhibition of effect on metastatic growth, other factors may have been involved, such as competitive inhibition by endogenous carbohydrate or lectins in the murine model. Furthermore, this emphasises the need to determine which HPA-binding glycoconjugate(s) are involved in this metastatic cell behaviour.

The study of metastasis mechanisms involves several inherent difficulties. Thus, the end point *in vivo* is the appearance of manifest metastatic foci, the development of which is influenced by a multitude of tumour cell- and host-associated factors. Injecting the tumour cells by the i.v. route limits the number of factors involved, but unfortunately it also reduces the similarity of the models to the situation in patients. In support of the biological relevance of the distinct tissue-preferenced growth seen in the present experiments, we have with LOX and FEMX-I cells observed similar tissue-preferenced metastasis development also after injection of the tumour cells either intrasplenic, i.p. or in the footpad of mice (Fodstad *et al.*, 1988*a*). In the same study we also showed that trypsinisation of the LOX cells before i.v. injection reduces their potential for forming lung colonies in the recipient animals. That this could have an effect on the HPA receptor was excluded in the present study, as trypsin treatment of intact cells did not alter the ability of LOX cells to bind HPA (data not shown).

In spite of the limitations of the experimental metastasis model used in this study, the data obtained are supported by studies in other model systems and in humans. Although it is inappropriate to make generalisations regarding cell characteristics involved in a complex process such as metastasis formation on the basis of correlations observed in one model system, our findings do support the recent retrospective studies in human cancers (Leathem & Brooks, 1987; Nishiyama *et al.*, 1987; Springer, 1989; Brooks & Leathem, 1991; Fukutomi *et al.*, 1991; Kakeji *et al.*, 1991; Schumacher *et al.*, 1992; Shiraishi *et al.*, 1992) implicating a role for HPA-binding glycoconjugates in site-specific metastasis development.

This work was supported by the Norwegian Cancer Society. We would like to thank Frances Jaques for secretarial assistance. We are also grateful to Dr Rosemary Walker for helpful discussions.

### References

ALTEVOGT, P., FOGEL, M., CHEINGSONG-POPOV, R., DENNIS, J., ROBINSON, P. & SCHIRRMACHER, V. (1983). Different patterns of lectin binding and cell surface sialylation detected on related high- and low-metastatic tumor lines. *Cancer Res.*, **43**, 5138–5144.

BROOKS, S.A. & LEATHEM, A.J. (1991). Prediction of lymph node involvement in breast cancer by detection of altered glycosylation in the primary tumour. *Lancet*, **338**, 71–74.

BUCKLEY, N.D. & CARLSEN, S.A. (1988). Involvement of soybean agglutinin binding cells in the lymphatic metastasis of the R3230AC rat mammary adenocarcinoma. *Cancer Res.*, **48**, 1451–1455.

CHEN, T.R. (1977). *In situ* detection of mycoplasma contamination in cell cultures by fluorescent Hoechst 33258 stain. *Exp. Cell Res.*, **104**, 255–262.

DENNIS, J., DONAGHUE, T., FLORIAN, M. & KERBEL, R.S. (1981). Apparent reversion of stable *in vitro* genetic markers detected in tumour cells from spontaneous metastases. *Nature*, **292**, 242–245.

DILLNER, M.L., HAMMARSTROM, S. & PERLMANN, P. (1975). The lack of mitogenic response of neuraminidase-treated and untreated human blood lymphocytes to divalent, hexavalent or insoluble *Helix pomatia* A hemagglutinin. *Exp. Cell Res.*, **96**, 374–383.

FINNE, J., CASTORI, S., FEIZI, T. & BURGER, M.M. (1989). Lectin-resistant variants and revertants of mouse melanoma cells: differential expression of a fucosylated cell-surface antigen and altered metastasizing capacity. *Int. J. Cancer*, **43**, 300–304.

FODSTAD, Ø., AASS, N. & PIHL, A. (1980). Assessment of tumour growth and of response to chemotherapy of human melanomas in athymic, nude mice. *Br. J. Cancer*, **41** (Suppl. IV), 146–149.

FODSTAD, Ø., HANSEN, C.T., CANNON, G.B., STATHAM, C.N., LICHTENSTEIN, G.R. & BOYD, M.R. (1984). Lack of correlation between natural killer activity and tumor growth control in nude mice with different immune defects. *Cancer Res.*, **44**, 4403–4408.

FODSTAD, Ø., BRØGGER, A., BRULAND, Ø., SOLHEIM, Ø.P., NESLAND, J.M. & PIHL, A. (1986). Characteristics of a cell line established from a patient with multiple osteosarcoma, appearing 13 years after treatment for bilateral retinoblastoma. *Int. J. Cancer*, **38**, 33–40.

FODSTAD, Ø., KJØNNIKSEN, I., AAMDAL, S., NESLAND, J.M., BOYD, M.R. & PIHL, A. (1988*a*). Extrapulmonary, tissue-specific metastasis formation in nude mice injected with FEMX-I human melanoma cells. *Cancer Res.*, **48**, 4382–4388.

FODSTAD, Ø., AAMDAL, S., MCMENAMIN, M., NESLAND, J.M. & PIHL, A. (1988*b*). A new experimental metastasis model in athymic nude mice, the human malignant melanoma LOX. *Int. J. Cancer*, **41**, 442–449.

FRAKER, P.J. & SPECK Jr, J.C. (1978). Protein and cell membrane iodination with a sparingly soluble chloramide, 1,3,4,6-tetrachloro-3,6-diphenylglucoluril. *Biochem. Biophys. Res. Commun.*, **80**, 849–857.

FUKUTOMI, T., HIROHASHI, S., TSUDA, H., NANASAWA, T., YAMAMOTO, H., ITABASHI, M. & SHIMOSATO, Y. (1991). The prognostic value of tumour associated carbohydrate structures correlated with gene amplifications in human breast carcinomas. *Jpn. J. Surg.*, **21**, 499–507.

GALEA, M.H., ELLIS, I.O., BELL, J., ELSTON, C.W. & BLAMEY, R.W. (1991). Prediction of lymph node involvement in breast cancer. *Lancet*, **338**, 392–393.

HAGMAR, B., ERKELL, L.J., BURNS, G. & RYD, W. (1990). Lectin binding in murine tumour lines with different malignant characteristics. *Invas. Metast.*, **10**, 317–328.

HAKOMORI, S. & KANNAGI, R. (1983). Glycosphingolipids as tumor-associated and differentiation markers. *J. Natl. Cancer Inst.*, **71**, 231–251.

ISHIKAWA, M. & KERBEL, R.S. (1989). Characterization of a metastasis-deficient lectin-resistant human melanoma mutant. *Int. J. Cancer*, **42**, 134–139.

KAKEJI, Y., TSUJITANI, S., MORI, M., MAEHARA, Y. & SUGIMACHI, K. (1991). Helix pomatia agglutinin activity is a predictor of survival time for patients with gastric carcinoma. *Cancer*, **68**, 2438–2442.

LEATHEM, A.J. & BROOKS, S.A. (1987). Predictive value of lectin binding on breast-cancer recurrence and survival. *Lancet*, **i**, 1054–1056.

NICOLSON, G.L. (1984). Cell surface molecules and tumor metastasis. Regulation of metastatic phenotypic diversity. *Exp. Cell Res.*, **150**, 3–22.

NISHIYAMA, T., MATSUMOTO, Y., WATANABE, H., FUJIWARA, M. & SATO, S. (1987). Detection of Tn antigen with *Vicia villosa* agglutinin in urinary bladder cancer: its relevance to the patient's clinical course. *J. Natl. Cancer Inst.*, **78**, 1113–1117.

PAULI, B.U., AUGUSTIN-VOSS, H.G., EL-SABBAN, M.E., JOHNSON, R.C. & HAMMER, D.A. (1990). Organ preference of metastasis. *Cancer Metast. Rev.*, **9**, 175–189.

READING, C.L. & HUTCHINS, J.T. (1985). Carbohydrate structure in tumor immunity. *Cancer Metast. Rev.*, **4**, 221–260.

READING, C.L., BELLONI, P.N. & NICHOLSON, G.L. (1990). Selection and *in vivo* properties of lectin-attachment variants of malignant murine fibrosarcoma cell lines. *J. Natl. Cancer Inst.*, **64**, 1241–1249.

RYE, P.D., DEARING, S.J. & WALKER, R.A. (1993). Carbohydrate profiles of primary breast carcinomas and their metastases. *Cancer J.*, **6**, 190–195.

SCHUMACHER, U., KRETZSCHMAR, H., BOOKS, S. & LEATHEM, A. (1992). Helix pomatia lectin binding pattern of brain metastases originating from breast cancers. *Pathol. Res. Pract.*, **188**, 284–286.

SHIRAISHI, T., ATSUMI, S. & YATANI, R. (1992). Comparative study of prostatic carcinoma bone metastasis among Japanese in Japan and Japanese Americans and whites in Hawaii. *Adv. Exp. Med. Biol.*, **324**, 7–16.

SPRINGER, G.F. (1989). Tn epitope (N-acetyl-D-galactosamine-O-serine/threonine) density in primary breast carcinoma: a functional predictor of aggressiveness. *Mol. Immunol.*, **26**, 1–5.

TAO, T.W. & BURGER, M.M. (1977). Non-metastasizing variants selected from metastasizing melanoma cells. *Nature*, **270**, 437–438.

TAYLOR, C.W., ANBAZHAGAN, R., JAYATILAKE, H., ADAMS, A., GUSTERSON, B.A., PRICE, C., GELBER, R.D. & GOLDHIRSCH, A. (1991). Helix pomatia in breast cancer. *Lancet*, **338**, 580.

WALKER, R.A. (1993). Helix pomatia and prognosis of breast cancer. *Br. J. Cancer*, **68**, 453–454.